# Differential impact in young and older individuals of blue-enriched white light on circadian physiology and alertness during sustained wakefulness

**DOI:** 10.1038/s41598-017-07060-8

**Published:** 2017-08-08

**Authors:** Virginie Gabel, Carolin F. Reichert, Micheline Maire, Christina Schmidt, Luc J. M. Schlangen, Vitaliy Kolodyazhniy, Corrado Garbazza, Christian Cajochen, Antoine U. Viola

**Affiliations:** 1 Centre for Chronobiology, Psychiatric Hospital of the University of Basel, 4012 Basel, Switzerland; 2 Transfaculty Research Platform Molecular and Cognitive Neurosciences, University of Basel, Basel, Switzerland; 3 Department of Psychiatry and Behavioral Sciences, Stanford University, Palo Alto, CA USA; 4 Cyclotron Research Center, University of Liège, Liège, Belgium; 5 Philips Lighting Research, High Tech Campus 36 5656AE, Eindhoven, The Netherlands; 6 Ziemer Ophthalmic Systems AG, 2562 Port, Switzerland; 7 PPRS, Paris, France

## Abstract

We tested the effect of different lights as a countermeasure against sleep-loss decrements in alertness, melatonin and cortisol profile, skin temperature and wrist motor activity in healthy young and older volunteers under extendend wakefulness. 26 young [mean (SE): 25.0 (0.6) y)] and 12 older participants [(mean (SE): 63.6 (1.3) y)] underwent 40-h of sustained wakefulness during 3 balanced crossover segments, once under dim light (DL: 8 lx), and once under either white light (WL: 250 lx, 2,800 K) or blue-enriched white light (BL: 250 lx, 9,000 K) exposure. Subjective sleepiness, melatonin and cortisol were assessed hourly. Skin temperature and wrist motor activity were continuously recorded. WL and BL induced an alerting response in both the older (*p* = 0.005) and the young participants (*p* = 0.021). The evening rise in melatonin was attentuated under both WL and BL only in the young. Cortisol levels were increased and activity levels decreased in the older compared to the young only under BL (*p* = 0.0003). Compared to the young, both proximal and distal skin temperatures were lower in older participants under all lighting conditions. Thus the color temperature of normal intensity lighting may have differential effects on circadian physiology in young and older individuals.

## Introduction

Human alertness and cognitive performance are regulated by a finely-tuned interaction of circadian and homeostatic processes that provide optimal and stable levels during a 16-h episode of normal wakefulness^1^. This stable recurrent daily pattern is ensured by the entrainment of endogenous human circadian rhythms to the 24-h light/dark cycle, the main “zeitgeber” (i.e. synchronizer) for this entrainment^2^. Besides the role of light as a zeitgeber, there is ample evidence that light has acute non-image-forming effects on human physiology, cognitive performance, alertness and well-being^3–6^. Light exposure suppresses evening and nighttime levels of the pineal hormone melatonin, increases or decreases cortisol expression depending on time of day^7–9^, and reduces subjective and objective correlates of sleepiness, particularly when administered in the late evening^10–12^. Studies investigating the impact of daytime light exposure on circadian physiology and alertness levels are less abundant and rather inconsistent^13–16^. Whether or not prolonged light exposure has sustained alerting, melatonin suppressing and cognitive performance-enhancing effects during extended wakefulness is not yet established, nor the influence of age.

With age, distinct alterations in sleep and cognition, as well as light perception, occur. These changes may originate at different systemic levels: at the input level (i.e., the eyes), the circadian pacemaker level (i.e., suprachiasmatic nuclei [SCN] in the hypothalamus) or downstream (i.e., specific brain regions). Since light perception changes with age, it is very likely that the eyes play a major role in possible associated circadian alterations. A number of age-related changes in the eye may contribute to reduced levels of light reaching the retina, such as (1) reduction in pupil size^17, 18^, (2) changes in spectral transmittance of the eyes’ crystalline lens and of ocular media density which attenuate short-wavelength light, thus modifying the spectral balance of broadband light at the retina^19, 20^, (3) yellowing of the human lens, which is a well-known age-related change of the ocular media^21^, (4) decreased transmittance of “ocular” light particulary in the short wavelength range^22^. Collectively, these changes may reduce the amount of entraining environmental light input to the circadian clock in the SCN^23^. Therefore, with age there could be an attenuated response to light exposure, which may potentially lead to desynchronization of circadian rhythms^24^ and could also be responsible for the earlier waking times and earlier circadian phases for core body temperature and melatonin rhythms observed in many older compared to young adults^25, 26^.

Little is known on whether improving lighting conditions (i.e., enhancing the zeitgeber stimulus) in older people may help to improve physiological degradation associated with age. Some researchers showed higher nighttime melatonin levels in older people after increasing daytime light intensity^27^, while others found no changes in healthy older people compared to young people^28^. These findings contradict the postulate that the decrease in melatonin levels is an age-related characteristic. Therefore, an investigation of the impact of light, in particular the role of blue-enriched light on sleepiness and circadian regulation of melatonin and cortisol in older people is of crucial importance: first because of our ageing society, and second, because of the growing availability of blue-enriched light sources due to new lighting technologies (e.g. LED)^29^.

Here we investigated the physiological and behavioural consequences of different normal indoor light intensity regimes on alertness, melatonin, cortisol, activity and core body temperature rhythms in young and older volunteers during 40 hours of sustained wakefulness under controlled conditions. We hypothesized that prolonged blue-enriched and non-blue-enriched white light exposure of 250 lx will enhance alertness levels in the young but not in the older participants when compared to dim light. We also hypothesized that circadian physiological responses will be differentially affected by the interplay of age and light exposure condition. We predicted an attenuation of melatonin secretion under blue-enriched as compared to non-blue enriched white light exposure in both age groups but less so in the older participants. Since to our best knowledge the effects of long-term light exposure (>24 h) on body temperatures and motor activity levels have not yet been investigated, we did not formulate an a priori hypothesis regarding these measures.

## Results

### Subjective sleepiness

**Figure 1:**
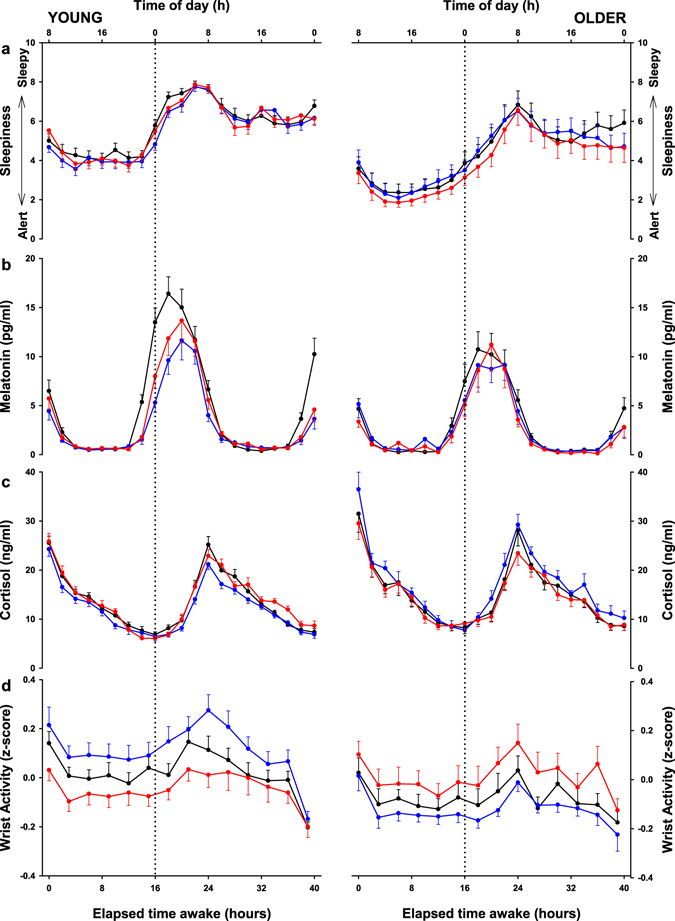
Time course of alertness, melatonin and cortisol profile and activity. Time course of the (**a**) Karolinska Sleepiness Scale (KSS), the (**b**) melatonin profile, the (**c**) cortisol profile and the (**d**) wrist activity in 26 young (left panel) and 12 older participants (right panel) under dim light (black lines), white light (red lines) or blue-enriched white light (blue lines). Data are plotted as a mean for each 2-hour bin for the KSS score, the melatonin and cortisol profile and in 3-hour bins for the wrist activity relative to elapsed time (hours) after wake-up. The error bars represent the standard error of the mean.

The time-course of subjective sleepiness is illustrated for each light condition and age group separately in Fig. 1a. The mixed model analysis yielded significance for the factors: “age”, “light condition” and “time of day” (Table 1).The older participants felt less sleepy during sustained wakefulness (40 h) compared to the young under all light exposure conditions (DL, WL and BL). Furthermore, irrespective of age, participants were less sleepy under both WL and BL compared to DL. The main effect of “time of day” and the interaction of “time of day x age” indicated the well- known circadian and homeostatic regulation of subjective sleepiness during sustained wakefulness with different dynamics in young and older participants, independent of the light conditions. Post-hoc comparisons, revealed that subjective sleepiness was lower in the older participants as compared to the young between 0 and 24-h (e.g. 08:00 to 08:00 h the next day) and between 32 and 40-h (e.g. 16:00 to 00:00 h) of elapsed time awake.

**Table 1:** Results of the analysis of variance for different physiological variables and subjective sleepiness over the time course of the study. In bold results with *p* < 0.05.

Analysis of variance							
Variable	Age	Light	Time of day	Age*Light	Age*Time	Light*Time	Light*Time*Age
KSS	**F**_**1,35.8**_ ** = 12.5**, ***p*** ** = 0.0011**	**F**_**2,1943**_ ** = 9.4**, ***p*** ** < 0.0001**	**F**_**20,1937**_ ** = 93.4**, ***p*** ** < 0.0001**	F_2,1943_ = 1.5, *p* = 0.2290	**F**_**20,1937**_ ** = 3.8**, ***p*** ** < 0.0001**	F_40,1937_ = 0.6, *p* = 0.9874	F_40,1937_ = 0.5, *p* = 0.9935
Melatonin	F_1,35.9_ = 2.7, *p* = 0.1070	**F**_**2,1943**_ ** = 14.2**, ***p*** ** < 0.0001**	**F**_**20,1926**_ ** = 106.2**, ***p*** ** < 0.0001**	**F**_**2,1943**_ ** = 5.2**, ***p*** ** = 0.0058**	**F**_**20,1926**_ ** = 2.4**, ***p*** ** = 0.0004**	**F**_**40,1926**_ ** = 2.2**, ***p*** ** < 0.0001**	F_40,1926_ = 0.7, *p* = 0.9007
Cortisol	**F**_**1,34.9**_ ** = 9.2**, ***p*** ** = 0.0046**	**F**_**2,1823**_ ** = 3.3**, ***p*** ** = 0.0357**	**F**_**20,1802**_ ** = 150.2**, ***p*** ** < 0.0001**	**F**_**2,1823**_ ** = 19.1**, ***p*** ** < 0.0001**	**F**_**20,1802**_ ** = 2.1**, ***p*** ** = 0.0026**	F_40,1802_ = 0.6, *p* = 0.9616	F_40,1802_ = 0.9, *p* = 0.6851
DPG	F_1,35.1_ = 2.7, *p* = 0.1088	**F**_**2,1189**_ ** = 15.8**, ***p*** ** < 0.0001**	**F**_**13,1183**_ ** = 94.2**, ***p*** ** < 0.0001**	**F**_**2,1189**_ ** = 9.8**, ***p*** ** < 0.0001**	F_13,1183_ = 1.3, *p* = 0.2075	F_26,1183_ = 1.0, *p* = 0.4328	F_26,1183_ = 0.2, *p* = 1.0000
Proximal temperature	**F**_**1,35.1**_ ** = 4.9**, ***p*** ** = 0.0330**	**F**_**2,1188**_ ** = 30.8**, ***p*** ** < 0.0001**	**F**_**13,1183**_ ** = 18.8**, ***p*** ** < 0.0001**	F_2,1188_ = 1.7, *p* = 0.1790	F_13,1183_ = 0.7, *p* = 0.7959	F_26,1183_ = 0.4, *p* = 0.9922	F_26,1183_ = 0.2, *p* = 1.0000
Distal temperature	**F**_**1,35.1**_ ** = 6.1**, ***p*** ** = 0.0183**	**F**_**2,1187**_ ** = 18.0**, ***p*** ** < 0.0001**	**F**_**13,1183**_ ** = 76.9**, ***p*** ** < 0.0001**	**F**_**2,1187**_ ** = 14.9**, ***p*** ** < 0.0001**	F_13,1183_ = 1.6, *p* = 0.0918	F_26,1183_ = 0.6, *p* = 0.9204	F_26,1183_ = 0.2, *p* = 1.0000
Wrist Activity	F_1,35.8_ = 3.4, *p* = 0.0735	F_2,1233_ = 2.4, *p* = 0.0896	**F**_**13,1224**_ ** = 14.9**, ***p*** ** < 0.0001**	**F**_**2,1233**_ ** = 37.0**, ***p*** ** < 0.0001**	F_13,1224_ = 1.6, *p* = 0.0904	F_26,1224_ = 0.3, *p* = 0.9994	F_26,1224_ = 0.5, *p* = 0.9909

KSS: Karolinska Sleepiness Scale; DPG: Distal Proximal Gradient.

### Salivary Melatonin and Cortisol

The well-known circadian profile of melatonin secretion was well-preserved in both age groups under all the three light conditions. We found a main effect of “time-of-day” and “light condition”, as well as an interaction of “light condition x age”, “time-of-day x age” and “light condition x time-of-day” (Table 1). Analyses by age group separately indicated, that all these effects were most likely driven by the young, as we found no significant differences between the light conditions in the older (light effect in the older: *p* = 0.2949), while young individuals exbited a significant attenuation of the evening increase in melatonin levels (light effect in the young: *p* < 0.0001) (Fig. 1b). Melatonin levels in the younger were suppressed under WL and BL from 14 to 18-h of elapsed time awake (e.g. 22:00 to 02:00 h) as compared to DL and even more so under BL than WL from 16 to 20-h of elapsed time awake (e.g. 00:00 to 04:00 h). The main effect of “light condition” for the evening melatonin onset (Table 2) yielded significance in both age groups, such that, when analysed separately, melatonin onset was delayed under BL (Young *p* < 0.0001; Older *p* = 0.0428) and WL (Young *p* < 0.0001; Older *p* = 0.0234) as compared to DL in both age groups. No significant differences were observed for the melatonin offset for any light treatment and age group (Table 2). A main effect of “light condition” was also found for the area under the curve, which appeared to be mainly driven by the young (light effect in the young: p = 0.0001), as no significant differences between the light conditions were observed in older individuals (light effect in the older: p = 0.1353), when analysed by age group seperately. Post-hoc comparisons yielded greater AUC in the young as compared to the older (*p* = 0.05) under DL and a smaller AUC under BL (*p* < 0.0001) and WL (*p* = 0.0007) compared to DL only in the young but not the older group. Similarly, the melatonin midpoint and melatonin midpoint by centre of gravity of the fitted curve (COG) yielded a main effect of “light condition” (Table 2), where timings were significantly delayed under BL (midpoint *p* = 0.0021; COG *p* < 0.0001) and WL (midpoint *p* = 0.0053; COG *p* = 0.0002) as compared to DL, only in the young group, when analysed by age group separately.

**Table 2:** Results of the analysis of variance for different melatonin variables. In bold results with *p* < 0.05.

Variable	Mean ± standard error of the mean			Analysis of variance		
	DL (Young/Older)	WL (Young/Older)	BL (Young/Older)	Light	Age	Light*Age
Mel-on (h:min ± h:min)	21:48 ± 00:12/22:10 ± 00:10	22:58 ± 00:11/22:54 ± 00:26	23:08 ± 00:20/22:40 ± 00:24	**F**_**2,58.7**_ ** = 17.1**, **p < 0.0001**	F_1, 35.4_ = 0.0, p = 0.9801	F_2,58.7_ = 1.5, p = 0.2417
Mel-off (h:min ± h:min)	07:55 ± 00:15/07:53 ± 00:25	07:52 ± 00:15/07:42 ± 00:28	07:42 ± 00:10/07:42 ± 00:13	F_2,60_ = 0.3 p = 0.7377	F_1,35.1_ = 0.3, p = 0.8647	F_2,60_ = 0.0, p = 0.9917
Midpoint (h:min ± h:min)	02:50 ± 00:13/03:01 ± 00:18	03:25 ± 00:11/03:17 ± 00:20	03:25 ± 00:12/03:11 ± 00:15	**F**_**2,58.1**_ ** = 4.9**, **P = 0.0107**	F_1,35.3_ = 0.0, p = 0.9469	F_2,58.1_ = 0.7, p = 0.5221
Amplitude (pg/ml)	17.23 ± 1.83/11.87 ± 2.07	14.29 ± 1.86/11.48 ± 2.40	12.32 ± 1.77/11.83 ± 2.09	F_2,57.5_ = 2.0, p = 0.1446	F_1,35_ = 1.4, p = 0.2505	F_2,57.5_ = 1.6, p = 0.2079
AUC (ng-hours/L)	5.47 ± 0.59/3.74 ± 0.65	4.04 ± 0.57/3.10 ± 0.68	3.25 ± 0.46/3.33 ± 0.62	**F**_**2,57**_ ** = 8.5**, **p = 0.0006**	F_1,34.8_ = 1.4, p = 0.2475	F_2,57_ = 2.4, p = 0.0974
COG (h:min ± h:min)	02:38 ± 00:13/02:56 ± 00:20	03:22 ± 00:12/03:17 ± 00:19	03:27 ± 00:12/03:11 ± 00:20	**F**_**2,58**_ ** = 9.2**, **p = 0.0003**	F_1,35.3_ = 0.0, p = 0.9726	F_2,58_ = 1.5, p = 0.2416

Mel-on: Melatonin onset; Mel-off: Melatonin offset; AUC: Area Under the Curve; COG: Centre Of Gravity of the fitted melatonin curve.

**Figure 2:**
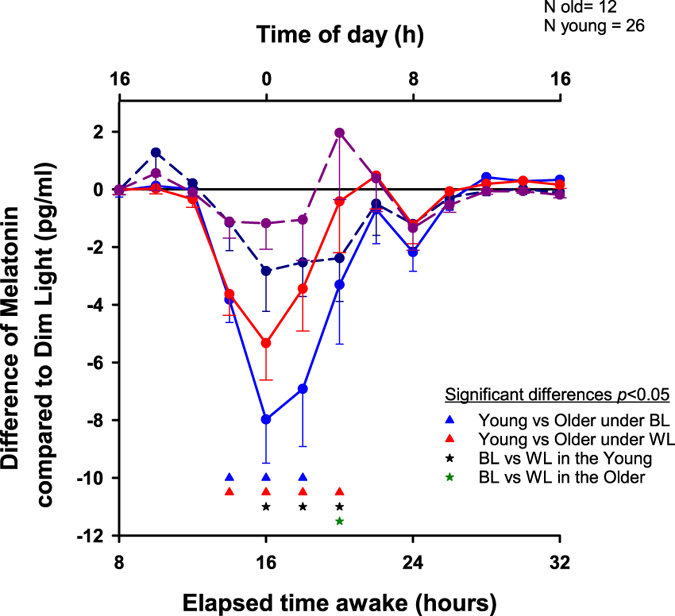
Time course of the change in melatonin concentration. Time course of the difference in melatonin concentration between the light conditions and DL (in 2 h bins). The horizontal black line represents the baseline under DL. The difference in melatonin concentration between BL and DL is depicted in light blue in the young (solid line) and in dark blue in the older (dashed line), and the difference between WL and DL is in red in the young (solid line) and in violet in the older (dashed line).

When melatonin levels under WL and BL are expressed as a difference to DL, a main effect of “time- of-day” (F_12,708_ = 11.08, *p* < 0.0001), “age x time-of-day” (F_12,708_ = 3.07, *p* = 0.0003) and “light Condition x time-of-day” (F_12,708_ = 1.85, *p* = 0.0378) is found, such that WL and BL suppressed more melatonin in the young as compared to older individuals (Fig. 2). While light exposure did not suppress melatonin in the older, it did for young during the first part of the night (e.g. 00:00 to 04:00 h) with a tendency to be more pronounced under BL compared to white light.

Cortisol profiles during 40-h of prolonged light exposure under sustained wakefulness revealed a clear circadian secretion pattern, with a peak of secretion in the morning, for both age groups under all three light conditions (Fig. 1c). We found a main effect of “age”, “time-of-day”, “light condition”, “age x time-of-day” and “age x light” (Table 1). Only in older individuals was cortisol significantly increased under BL compared to WL (*p* = 0.04) and also tended to be higher compared to DL (*p* = 0.08). Cortisol profiles did not show significant differences between young and older participants under DL (*p* = 0.18) and WL (*p* = 0.38) exposure. However, under BL exposure older participants had higher cortisol levels than the young (*p* = 0.0003, Table 1).

### Wrist activity

Wrist motor activity levels yielded a significant main effect of “time-of-day” (Table 1), such that activity levels were stable during the daytime and increased during the biological night (Fig. 1d). This pattern was observed in both age groups and under all light conditions. The interaction of “light x age” was significantly different (Table 1). Post-hoc comparisons revealed a tendency for increase in activity levels under BL as compared to DL (*p* = 0.0750) and a significant decrease of activity under WL (*p* = 0.0115) in the young, while in the older a decrease was observed under BL as compared to WL (*p* = 0.0358). Interestingly, no differences were found for the level of activity between the young and the older under WL (*p* = 0.7134) and DL (p = 0.1396), although older individuals showed lower activity levels as compared to the young under BL (*p* = 0.0025).

### Skin temperature

**Figure 3:**
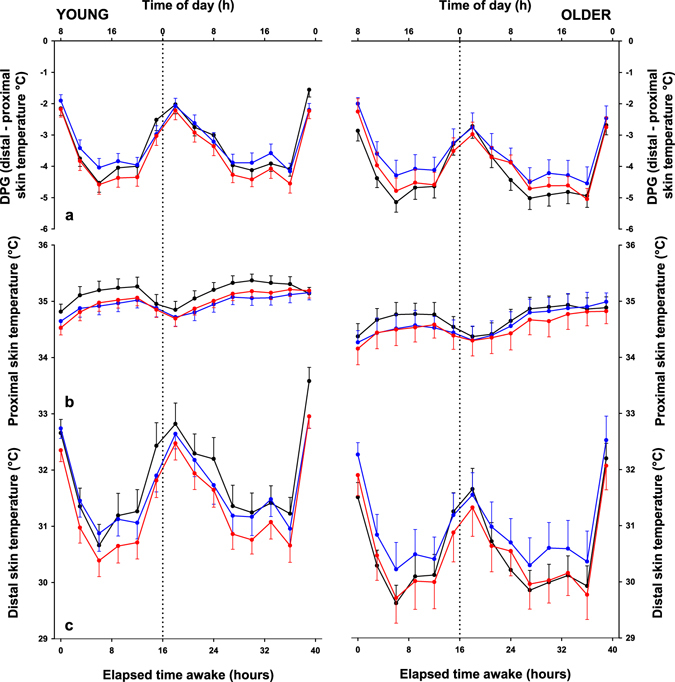
Time course of the distal-to-proximal skin temperature gradient (DPG), proximal and distal skin temperatures. Time course of the (**a**) distal-to-proximal skin temperature gradient (DPG), (**b**) proximal skin temperature and (**c**) distal skin temperature in 26 young participants (left panel) and 12 older (right panel) under dim light (black lines), white light (red lines) or blue enriched white light (blue lines). Data are plotted as a mean for each 3-hour bin relative to elapsed time (hours) after wake-up. The error bars represent the standard error of the mean.

Distal temperature (DT) showed maximal values during the night and minimal values during the day. We observed a rapid decrease in the early morning hours (from 1:00 to 10:00 h), and a rapid rise during the night (from 18:00 to 1:00 h) for both age groups. Light exposure significantly impacted on DT in a age-dependent manner (Fig. 3c). Thus, the factors “age”, “light condition”, “time-of-day” and the interaction “light condition x age” yielded significance (Table 1). Post-hoc comparisons revealed a significant decrease in DT in the older as compared to the young under DL (*p* = 0.0047) and WL (*p* = 0.05).

In contrast, proximal skin temperature (PT) showed maximal values during the day and minimal values during the night. Main effects of “light”, “age” and “time-of-day” were significantly different (Table 1 and Fig. 3b). Post-hoc comparisons revealed a significant decrease of PT under WL as compared to DL (*p* = 0.0423) irrespective of age. Furthermore, PT was higher in the young as compared to older participants across all light settings (*p* = 0.0357).

The distal-to-proximal skin temperature gradient (DPG) exhibited a similar temporal profile as for DT but with larger amplitude. Main effects of “time-of-day”, “light” and the interaction “age x light” were significantly different (Table 1 and Fig. 3a). Post-hoc analysis revealed that older participants have lower DPG levels as compared to the young even under DL (p = 0.0341). Furthermore, only the older participants showed an increase in DPG under BL (*p* = 0.0575) compared to DL.

## Discussion

Compared to dim light, moderate 250 lx (an intensity usually used indoors) white light or blue-enriched white light significantly impacts on sleepiness, melatonin and cortisol profile, skin temperatures and motor activity in an age-dependent manner in healthy volunteers.

### Does extended light exposure modify circadian markers in the young?

Many studies have shown that bright light exposure suppressed melatonin levels or shifted its circadian profile^30–33^. These non-visual light responses were more pronounced under light at short wavelengths^4, 11, 34–36^, which is in line with the greater attenuation of the evening increase in melatonin we found under BL in the young. The melanopic content of the light source was 22 times greater under BL than WL, and 333 times greater than DL. Thus, the melanopsin-containing intrinsic photosensitive retinal ganglion cells (ipRGCs) were activated more under BL than under WL and DL, even though we cannot exclude the participation of rods and cones^37, 38^. These ipRGCs are connected to the SCN through the specialized non-image-forming retinohypothalamic tract. As the pineal gland receives indirect neuronal projections from the SCN^39^, phase shifting of the nocturnal melatonin rhythm provides a marker for the effect of light on the circadian system - the classical phase response curve (PRC)^40^. Attenuation by light of the evening rise in melatonin can be explained by an acute effect of evening light and the induction of a phase delay. However, we do not have unequivocal evidence for a phase delay, since the timing of the melatonin offset was not shifted. This may be due to the fact that study participants were continually exposed to light during the phase advance portion of the PRC in the second half of the night. Thus, the probable phase delay caused by evening light was counteracted by a phase advance in the early morning, resulting in no phase shift of offset. Furthermore, the attenuation of the evening increase in melatonin in the young occurred only in the first half of the subjective night. In the second part of the night, beyond 20 hours of light exposure, no melatonin suppression occurred for both 250 lx light conditions. 36 hours after the start of the sustained light exposure, in the late evening of the second day of the 40-h protocol, melatonin was again suppressed. Thus, the suppressing effect of constant light exposure was only present during active melatonin secretion but not after the offset of melatonin synthesis (between 3 to 6 am) resulting in a reduced nighttime AUC of melatonin. Regarding the phase shifting effects of continuous light exposure, our results also support the two oscillator model^41–43^ of a morning oscillator (M) entrained by the dawn light signal and an evening oscillator (E) entrained by the dusk signal. Our observed “null shift in circadian phase” during 40-h of continuous light exposure could be the result of a “net shift” of a phase delay of the E oscillator and phase advance of the M oscillator. In addition, the alerting response to light was predominantly present in the evening together with the attenuation of the rise of melatonin. This is in accordance with previous studies, which showed that melatonin initiates also sleepiness, acting therefore as the hormonal trigger between body heat loss and induction of sleep in the evening (opening of the sleep gate)^44–47^.

Some studies have shown that the normal transition from sleep to wakefulness, as well as switching on the light in the morning^7^, amplify the morning rise in cortisol secretion. Sleep deprivation also increased cortisol levels^48, 49^ but reduced the cortisol awakening response. Bright light exposure on the rising and descending phases of the cortisol rhythm (i.e., when cortisol levels are high) decreased cortisol levels, however no effect was found when light was administered at the end of the descending phase^8, 9^, which supports the hypothesis that the effects of light on cortisol depend on the circadian phase at which the exposure occurs^50, 51^. Whereas a 10,000 lx light exposure decreased cortisol levels, other studies reported no change with nighttime and early morning 600 to 3,000 lx broad spectrum light exposure between 20-h and 6-h^12, 52–54^, or extended nighttime 100 or 1,000 lx light exposure between 18-h and 8-h^55^. The influence of light on cortisol levels may depend on light intensity, which would be consistent with the dose-response curves observed for melatonin suppression^56^, although the exact dose-relationship for light illuminance and cortisol changes is not yet known. Our data in the young participants are partly in accordance with these findings, such that a long term exposure to moderately bright light (250 lx) decreased cortisol levels under BL compared to DL and WL.

Skin temperatures play a major role in the regulation of the endogenous circadian core body temperature (CBT) rhythm. As previously described, distal skin temperatures show an inverse and higher amplitude rhythm than proximal skin temperature and CBT^57, 58^. This opposite pattern of distal and proximal skin temperature rhythms reflects the differences in thermophysiological regulatory mechanisms. There is evidence, that a rostral projection from the circadian pacemaker to the preoptic areas serves the circadian modulation of CBT^59^ and that all these autonomically regulated mechanisms of shell size occur through constriction or dilatation of blood vessels (arterioles and arteriovenous anastomosis) in distal skin regions^60^. Indeed, when the arteriovenous anastomoses are open, blood goes rapidly from the core to the distal skin regions, leading to body heat loss and decrease in CBT. The distal-proximal skin temperature gradient (DPG) provides a selective measure of distal skin blood flow, and, hence, of efficient body heat loss via the extremities^61, 62^. It has previously been shown that these modulations in DPG are associated with melatonin secretion onset^63, 64^, such that melatonin selectively augments distal skin blood flow while leaving proximal skin blood flow and cerebral blood flow unaffected^65^. Nevertheless, here we did not find a temporal correlation of light induced changes in evening melatonin secretion and corresponding changes in skin temperatures.

### Does extended light exposure have the same effect on circadian markers in the older as compared to young?

Normally, lens absorption is more pronounced for short wavelength light that is optimal for appropriate entrainment of the endogenous biological clock^19^. With ageing, the lens increases in density^20, 21, 66^, causing an alteration in the spectral absorption, and darkens with development of a yellow pigmentation that further reduces light transmission to the retina^67^. As a result, the level of light, particularly blue wavelengths, reaching the retina will be reduced, leading to a weaker photic entrainment of the circadian clock. This could explain some changes in our older volunteers, such as the weaker melatonin suppression by light. Furthermore, even though the peak sensitivity in older people is shifted from 484 nm to 494 nm, no significant differences were found for phase shifts and melatonin suppression under short wavelength light exposure^68, 69^. There was still a small attenuation in the evening melatonin secretion in the older as found in the young group. There is recent evidence that increased lens filtering does not necessarily lead to decreased non-visual sensitivity to light, due to as yet undefined adaptive mechanisms^66^, such as dendritic sprouting in different retinal layers^70^, regulation of melanopsin mRNA and protein expression by environmental illumination^71, 72^, or long-term alteration of light history that could lead to an increased light sensitivity. Thus, the increase in lens density with age may not be the only explanation for the observed attenuated melatonin response by light in our older participants.

Alternatively, due to the loss of ganglion cells with age^73^, older subjects may need higher light intensities to achieve the same light response as the young^74^. However, melatonin levels under dim light were already at a rather low level in this group, which is in accordance with other studies^75, 76^. Thus, the intensity of the light exposure in this study may not have been strong enough to suppress melatonin concentration any further. Indeed, even though some recent studies did not show an age related effect on melatonin responses to different light intensities over 1,000 lx^77, 78^, light exposure between 100 and 1,000 lx did have a weaker effect on melatonin phase shifts in older participants compared to the young^24^.

Several laboratory studies have shown that non-visual responses to light depend on preceding light history^79–84^. In general, the non-visual response to a light exposure is larger when the preceding light exposure is less bright: people coming from a dimly lit environment respond more strongly to light than people coming from a brightly lit environment. Thus, it could be that 250 lx constant light during previous daytime leads to less suppression in the following night. Daytime light exposure could even increase nocturnal melatonin secretion^27^.

Additionally, sleep deprivation is known to reduce light responsiveness of the SCN by lowering circadian phase-shifting capacity^85^ and decreasing SCN neuronal activity^86, 87^. Thus, both light history and sleep deprivation may account for the rather limited melatonin suppression by constant light, and for the unchanged circadian markers in the study.

Accordingly, for both age groups, the amount of sleep, and the preceding light exposure on the day/evening before (or even during) the night are relevant to consider in regard to light sensitivity changes.

Surprisingly we found an increase in cortisol levels in the older group under BL compared to WL and DL and compared to the young group under BL as well. These data can neither be explained by a higher stress during the study in the older participants as they were less tense and felt more comfortable and happy compared to the young (data not shown), nor by greater activity as they moved less under BL compared to the young. So far, we do not have an explanation for this cortisol variation as it is the first time that people were exposed to a continuous blue- and non-blue enriched light for 40-h. The only extended nighttime light exposure (from 22-h to 8-h) at 100 or 1000 lx reported no significant differences in cortisol profile^55^.

Additionally, we discovered that skin temperature was generally lower in the older compared to the young. One can argue that because older people accumulate more fat than the young, as shown by a higher body mass index (BMI), and that a high fat content creates an insulating barrier for conduction and exchange of heat^88^, they will exhibit decreased heat dissipation in proximal body areas. However, we also found lower skin temperature in distal body areas in the older, which can not be explained by a higher BMI. Alternatively, there is evidence that endothelin-1 mediated vasoconstrictor tone is greater in older compared with young men^89^, which could explain generally lower skin temperature levels in our older participants.

Moreover, we found an increase in distal temperature under BL in the older with no change under WL compared to DL. This finding needs further investigation in order to understand the underlying mechanism, also taking into account other variables implicated in human thermoregulation.

A higher BMI has also been shown to be correlated with lower physical activity^90^, which is in line with our results presenting a global lower or equal activity in the older compared to the young. Nevertheless, we have no explanation for the age-dependent light effect on activity levels in our study.

However, the global shape of motor activity across all groups and conditions is in accordance with the circadian modulation of the other variables; the maximum of activity was reached at the maximum of cortisol levels (circadian cortisol peak), when participants were most sleepy, in the descending phase of melatonin concentration. One explanation for this activity increase is a compensatory behaviour to possibly counteract circadian sleep promotion and high sleep pressure levels during this time window.

## Conclusion

In this study, circadian variations of physiological variables were maintained during 40-h of extended wakefulness in both age groups, independent of light treatments. Furthermore, even though the older felt less sleepy than the young under the dim light condition, light had a significant alerting response in both age groups during the active melatonin secretory phase, which corresponds to the late evening and the first part of the night. In young participants evening and nighttime melatonin levels were attenuated under both light conditions during the late evening and the first part of the subjective night only, but not in the remainder of the night - an effect which was less pronounced in the older and not reaching significance. Further investigations are needed to understand the age-dependent light effect on cortisol, skin temperature and wrist activity.

As typical daylight exposure outdoors is in the order of tens of thousands lx, artificial light exposure indoors is relatively modest and limited to a few hundred lx only. Therefore, people who depend on artificial light, such as night- or shift-workers, as well as older and nursing home residents, will suffer from lack of light, particularly in the winter months. Thus, the use of moderately bright light in night work, shift work settings and nursing homes, where constant light levels are very common, may have differential effects on young and older people. Here we have evidence that prolonged moderate light levels at 250 lx decrease overall melatonin secretion during the night, particularly in young people, without affecting circadian phase, while both age groups felt more alert under blue and non-blue enriched light during the late evening and first half of the night. Therefore, lighting conditions and installations need to be adapted in intensity and spectrum depending on the age of the targeted population.

## Materials and Methods

### Ethical Approval

All participants gave written informed consent. The study protocol, screening questionnaires and consent forms were approved by the local ethics committee (EKBB/Ethikkommission beider Basel, Switzerland), and conformed to the Declaration of Helsinki.

### Study participants

Study volunteers were recruited through advertisements at different universities and internet sites in the region. The screening procedure began with a telephone interview. Then all participants completed an informed consent form, a General Medical Questionnaire, the Beck Depression Inventory II (BDI-II)^91^, the Epworth Sleepiness Scale (ESS)^92^, the Horne Ostberg Morningness Eveningness Questionnaire (MEQ)^93^, the Munich Chronotype Questionnaire (MCTQ)^94^ and the Pittsburgh Sleep Quality Index (PSQI)^95^. We excluded participants with general medical disorders, current or past psychiatric and sleep disorders and a usual sleep duration of less than seven or more than nine hours. PSQI values were required to be below 5 and BDI-II values below 12. Further exclusion criteria included smoking, medication (except oral contraceptives) and drug consumption. We excluded shift workers and applicants who had transmeridian flights in the three previous months. All women were tested for pregnancy. Women without hormonal contraceptive use were studied during the luteal phase of their menstrual cycle.

One week before the study, the volunteers were requested to abstain from excessive alcohol and caffeine consumption to prevent withdrawal effects. Furthermore, they were instructed to keep a regular sleep-wake schedule (8-h sleep at night and no daytime naps) to ensure stable circadian entrainment. Compliance to this outpatient segment of the study was verified using wrist actigraphs (Actiwatch L, Cambridge Neurotechnologies, Cambridge, UK) and self-reported sleep logs.

To rule out sleep disturbances and to assess their ability to sleep in a new environment, participants slept one night at the Centre for Chronobiology prior to study begin. They also underwent a medical screening to guarantee physical and mental health, as well as an ophthalmologic examination in order to exclude volunteers with visual impairments (visual field, colour vision, pupillary reflex).

Out of 650 potential participants, we selected 38 healthy volunteers who successfully fulfilled all criteria. The young group included 26 participants between 20 and 35 years (mean age (SE): 24.96 (0.58) years) and the older group comprised 12 participants between 55 and 75 years (mean age (SE): 63.58 (1.27) years). The two age groups did not differ according to sleep and wake times, BDI, ESSm and PSQI (Table 3). However, they differed in the BMI, the MEQ and the MCTQ, such that the older participants had a higher BMI compared to the young and were more morning types while the young were more intermediate chronotypes.

**Table 3:** Characteristics of the group of participants.

	YOUNG Mean (SE)	OLDER Mean (SE)	*p*
N (m, f)	26 (15,11)	12 (9,3)	
Age (year)	24.96 (0.58)	63.58 (1.27)	**<0.0001**
Sleep time (h: min)	23:23 (00:06)	23:10 (00:09)	0.2419
Wake time (h: min)	07:23 (00:06)	07:10 (00:09)	0.2419
BDI	2 (0.44)	1.83 (0.56)	0.8261
ESS	5.37 (0.60)	4.23 (0.66)	0.2559
MEQ	54.34 (1.37)	63.42 (3.13)	**0.0037**
MCTQ	4.71 (0.16)	2.86 (0.44)	**<0.0001**
PSQI	3.32 (0.31)	3 (0.46)	0.5635
BMI (kg/m^2^)	22.19 (0.49)	25.32 (0.73)	0.0010

BDI: Beck Depression Inventory; ESS: Epworth Sleepiness Scale; MEQ: Horne & Ostberg morningness eveningness Questionnaire; MCTQ: Munich Chronotype Questionnaire; PSQI: Pittsburgh Sleep Quality Index; BMI: Body Mass Index.

### Laboratory part

**Figure 4:**
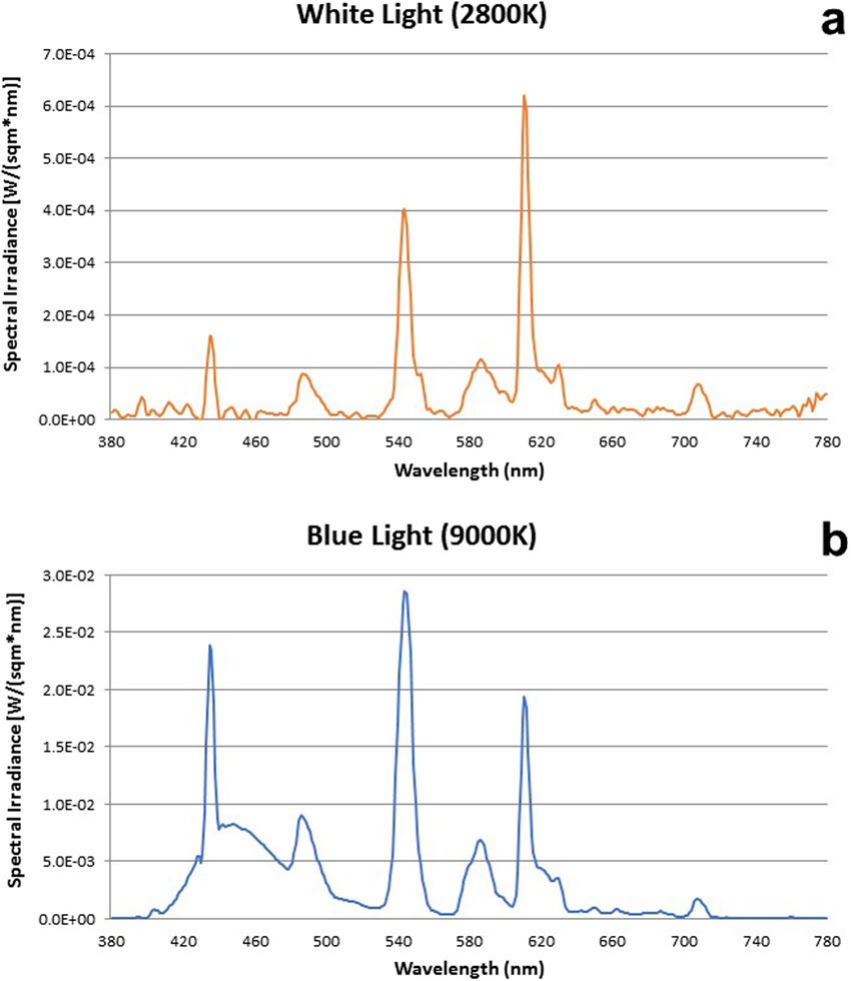
Spectral composition. Spectral composition (light wavelength by irradiance; W/m^2^-nm) of the (**a**) polychromatic white light and the (**b**) blue-enriched polychromatic white light.

The study consisted of three segments [i.e. one control dim light condition and two experimental light conditions] of a 40-h sleep deprivation protocol performed in a balanced cross over design, separated by at least 3 weeks. Each segment lasted 56-h, starting with a baseline night of 8-h (according to subject’s habitual sleep time), followed by a 40-hour episode of scheduled wakefulness, and ended with an 8-h recovery night (according to individual habitual sleep time). Each participant remained in a windowless and sound-attenuated bedroom, without any time-of-day information. The light treatment of each condition consisted of 40-h fluorescent white light exposure starting at subject’s wake time, with either (a) control dim light (DL: <8 lx), (b) white light (WL: 250 lx, 2,800 K) or (c) blue enriched white light (BL: 250 lx, 9,000 K). All illuminances were vertical illuminances at the eye position of the participant (Fig. 4). This light system used two different fluorescent tubes provided by Philips (*Philips Research*, *Eindhoven*, *The Netherlands*); a 2,700 K lamp (*Master TL5 HO 54w/827*) and a 17,000 K lamp (*Master TL5 HO Activiva Active 54w 1sl*). The ambient reflections and optical conditions resulted in an effective colour temperature that deviated from these values. We used 2,800 K and 9,000 K since they represent daylight color temperatures we experience in the morning and evening (2800 K) and during midday under a blue sky (9000 K).The melanopic lx was 101 under white light (intensity: 0.070 mW/cm^2^; photon density: 2.00417E+18 photons/m^2^s) and 271 melanopic lx under the blue-enriched white light (intensity: 0.087 mW/cm^2^; photon density: 2.30E+18 photons/m^2^s) according to Lucas *et al*.^96^. Participants were asked to take part in at least two conditions (control condition and an experimental condition: BL-DL: five young (4 m/1f) and one older (1 m/0f)/WL-DL: six young (4 m/2f) and two older (1 m/1f)) and, if they wanted, could complete the third condition (experimental condition: BL-DL-WL: 15 young (7 m/8f) and nine older (7 m/2f)). All conditions were controlled with regard to light influence (no light-emitting electronic devices such as smartphones and internet were allowed), caloric intake (standardized meals every two hours), and body posture (semi-recumbent position during scheduled wakefulness). Participant’s movements were reduced to a minimum and they had to stand-up for regularly scheduled computer tests (light screen <10 lx) and bathroom visits. Social interaction for participants was restricted to the contact with examiners and study helpers. All participants were allowed to carry out the following activities during all lighting conditions: reading, listening to music, writing or drawing, knitting, doing puzzles, and talking to the study helpers. They did not have access to smartphones and the internet as well as to other light emitting devices except for the monitor of the testing computer.

### Assessment of subjective sleepiness

Subjective sleepiness was assessed every hour throughout scheduled wakefulness, using the Karolinska Sleepiness Scale (KSS)^97^.

### Salivary melatonin and cortisol

Saliva samples were collected at regular intervals during scheduled wakefulness to assess melatonin and cortisol levels. Sampling rates dynamically changed with circadian phase, such that sampling frequency was decreased during the biological day when melatonin secretion is low (one sample every hour), and increased during the biological evening, night and early morning hours (one sample every 30 minutes)^98^. Salivary samples were immediately frozen and kept at −20 °C until the melatonin and cortisol assays were conducted.

A direct double-antibody radioimmunoassay was used for the melatonin assay [validated by gas chromatography–mass spectroscopy with an analytical least detectable dose of 0.65 pg/mL; Bühlmann Laboratories, Schönenbuch, Switzerland; ref. 99]. The minimum detectable dose of melatonin (analytical sensitivity) was determined to be 2 pg/mL and the interassay coefficients of variation (CVs) were 20.1% at 0.60 pg/ml, 2.6% at 7.24 pg/ml, and 4.8% at 24.42 pg/ml^99^.

Cortisol was measured by ALPCO (ALPCO Diagnostics, Salem, NH, USA), using a direct salivary enzyme-linked immunosorbent assay (ELISA) for quantitative determination of cortisol. The sensitivity was 1.0 ng/ml and the intra-assay coefficient of variances amounts to 10.3% for baseline values 6.6 ng/ml.

### Wrist activity

Rest–activity cycles were measured by wrist actimetry using the Actiwatch system (Cambridge Neurotechnology Ltd, UK). Participants continuously wore the actimeter on the non-dominant hand during each 56-h study segment. Actimetry data were analysed by the Sleep and Activity Analysis Software 7.23 V (Cambridge Neurotechnology Ltd, UK). To avoid device-related sensitivity differences, the activity data were normalized for each actiwatch and averaged per 3-h epoch.

### Skin temperature

Skin temperature was recorded throughout the 56-h of the protocol at a rate of one sample per minute. Recording ended immediately after the recovery night. Skin temperature was measured using wireless temperature sensors (DS1922L, Thermochron, iButtons®, Maxim Integrated Products, Sunnyvale, CA, USA; resolution 0.0625 °C; see ref. 100). The iButtons® were fixed to the skin with thin, air-permeable adhesive surgical tape on 6 locations: both hands (ventral part of left and right wrist), both feet (inner part of left and right foot, just below the ankle bone) and left and right infraclavicular region. For the analysis, distal skin temperature (DT) was calculated by averaging the skin temperature of both hands and feet, and proximal skin temperature (PT) was calculated as the average temperature of the left and right infraclavicular region. The distal-to-proximal skin temperature gradient (DPG) was defined as the difference between the proximal and distal skin temperatures (DT-PT)^101^.

### Statistical analysis

For all analysis, if not stated otherwise, the statistical package SAS (version 9.1; SAS Institute, Cary, NC, USA) was used with a mixed-model analysis of variance for repeated measures (PROC MIXED) with within factors “age” (young [Y] versus older [O]), “light condition” (dim light [DL] versus blue-enriched white light [BL] versus white light [WL]) and “time-of-day” (all assessed time points) and two random factors “subject” and “order of the session”. In order to reduce short-term fluctuations and the number of time segments, continuously recorded data were averaged in 3-h blocks. Contrasts were assessed with the LSMEANS statement and p values were based on Kenward-Roger’s corrected degrees of freedom^102^. We also ran the same analysis for each age group separately, with the factors “light condition” and “time-of-day”.

All time-course measures were expressed as elapsed time awake according to each participant’s wake-up time. Data are plotted relative to elapsed time awake and the average time-of-day indication was also added for visual clarity. Time-of-day was calculated relative to the mean of the participant’s usual wake-up time, as they did not have the same scheduled time. All analyses comprised 26 young and 12 older participants, except for the cortisol and temperature assessment where only 25 young were involved (one participant did not provide enough saliva samples to assess cortisol profile, and the iButtons of another participant did not record skin temperature during the study).

For the melatonin onset and offset (Mel-on and Mel-off) following exposure to either DL, WL or BL, the melatonin data were resampled every minute using linear interpolation and the analysis was based on the mid-range crossing (25% of the amplitude) of bimodal skewed baseline cosine function^103^ fitted to the resampled data as in ref. 104. The midpoint time was also calculated as the average time between the Mel-on and Mel-off. Melatonin amplitude was defined as the difference of the peak level to baseline levels of the bimodal skewed baseline cosine function (BSBCF) curve^103^, the area under the curve encompassed the region from the baseline to the peak and “COG” time. In the present manuscript, a modified cost function J(MEL, BSBCF) = MeanSE(MEL, BSBCF)/Var(BSBCF) was used, where “MEL”, “BSBCF”, “MeanSE”, and “Var” were the resampled mealonin data, values of the BSBCF function, mean squared diffference, and variance, respectively. The modified function J(MEL, BSBCF) more efficiently avoids the trivial solution with all values of the fitted curve equal to zero for melatonin data with narrow peaks. For these analyses only 25 young were involved due to missing values around the peak for one participant.

Melatonin suppression was also assessed at the different time points during the night. The difference of melatonin variation under BL and WL compared to DL was calculated for each subject and for each lighting condition. Then these values were averaged and plotted in a graph as a function of time. This analysis was done over a 24-h melatonin profile, from 16:00 h on the first day to 16:00 h on the second day after the sleep deprivation night.
